# In vitro antimicrobial activity of Medilox® super-oxidized water

**DOI:** 10.1186/1476-0711-13-29

**Published:** 2014-07-14

**Authors:** Murat Gunaydin, Saban Esen, Adil Karadag, Nevzat Unal, Keramettin Yanik, Hakan Odabasi, Asuman Birinci

**Affiliations:** 1 Department of Medical Microbiology, Istanbul University, Cerrahpasa Medical Faculty, Istanbul, Turkey; 2 Department of Infectious Diseases, Ondokuz Mayis University Faculty of Medicine, Samsun, Turkey; 3 Department of Medical Microbiology, Ondokuz Mayis University Faculty of Medicine, Samsun, Turkey; 4 Samsun Maternity and Children's Hospital Laboratory of Microbiology, Samsun, Turkey

**Keywords:** Super-oxidized water, Medilox, Disinfectant, Bacteria, Fungi

## Abstract

**Aim:**

Super-oxidized water is one of the broad spectrum disinfectants, which was introduced recently. There are many researches to find reliable chemicals which are effective, inexpensive, easy to obtain and use, and effective for disinfection of microorganisms leading hospital infections. Antimicrobial activity of super-oxidized water is promising. The aim of this study was to investigate the in-vitro antimicrobial activity of different concentrations of Medilox® super-oxidized water that is approved by the Food and Drug Administration (FDA) as high level disinfectant.

**Material and methods:**

In this study, super-oxidized water obtained from Medilox® [Soosan E & C, Korea] device, which had been already installed in our hospital, was used. Antimicrobial activities of different concentrations of super-oxidized water (1/1, 1/2, 1/5, 1/10, 1/20, 1/50, 1/100) at different exposure times (1, 2, 5, 10, 30 min) against six ATCC strains, eight antibiotic resistant bacteria, yeasts and molds were evaluated using qualitative suspension test. Dey-Engley Neutralizing Broth [Sigma-Aldrich, USA] was used as neutralizing agent.

**Results:**

Medilox® was found to be effective against all standard strains (*Acinetobacter baumannii* 19606, *Escherichia coli* 25922, *Enterococcus faecalis* 29212, *Klebsiella pneumoniae* 254988, *Pseudomonas aeruginosa* 27853, *Staphylococcus aureus* 29213), all clinical isolates (*Acinetobacter baumannii*, *Escherichia coli*, vancomycin-resistant *Enterococcus faecium*, *Klebsiella pneumoniae*, *Pseudomonas aeruginosa*, methicillin-resistant *Staphylococcus aureus*, *Bacillus subtilis*, *Myroides* spp.), and all yeastsat 1/1 dilution in ≥ 1 minute. It was found to be effective on *Aspergillus flavus* at 1/1 dilution in ≥ 2 minutes and on certain molds in ≥ 5 minutes.

**Conclusion:**

Medilox® super-oxidized water is a broad spectrum, on-site producible disinfectant, which is effective on bacteria and fungi and can be used for the control of nosocomial infection.

## Introduction

Disinfection is elimination of pathogen microorganisms except spores of spore-forming bacteria on inanimate medical supplies using chemicals called disinfectants [1]. Disinfectants and antiseptics are required for infection control in hospitals [2]. It has been known that the removal of these microorganisms from the hospital environment prevents the occurrence of many infections [3]. Microorganisms causing nosocomial infections have been varied in time due to various factors. In the last 25-30 years, fungi have been increased significantly besides bacterial agents. The most common fungal pathogens that cause hospital infections are *Candida albicans*, other *Candida* species and *Aspergillus* species [4-6]. One of the most important steps in the prevention of nosocomial infections is the selection of antiseptics and disinfectants which are effective against these microorganisms [7]. Activity spectrum, compatibility with surfaces, exposure time, cost, environmental effects, and damage to the medical instruments are considered for the selection of disinfectants in hospitals. However, disinfectants can harm human health and environment due to their physicochemical properties, toxic effects and waste problem. All these disadvantages should be kept in mind while choosing the right, inexpensive, easy to apply and reliable disinfectants for hospitals [1].

Super-oxidized water which is a widely used disinfectant in recent years, has many advantages such as not having toxic products, not harming human tissue, low cost, safety to the patients, the staff, and the environment [8]. Super-oxidized water is obtained by applying an electric current on salty water. Thanks to its broad spectrum against microorganisms it is used for disinfection and sterilization purposes. After electrolysis it comprises hypochlorous acid, hypochlorite ions, dissolved oxygen, ozone, superoxide radicals which has strong oxidation potential and shows strong antimicrobial activity. It can kill bacteria, viruses, fungi and parasites very fast and can be used for disinfection of hard surfaces and water systems [9].

Super oxidized water, which was introduced recently, is among the broad spectrum disinfectants with its promising antimicrobial activity on microorganisms. The aim of our study was to investigate the in-vitro activity of super-oxidized water at different concentrations against extended group of microorganisms including bacteria and fungi causing hospital-acquired infections.

## Materials and methods

In our study, activity of super-oxidized water, which was produced in Medilox® (Soosan E & C, Korea) device which had been already installed in Ondokuzmayis University, on different types of bacteria and fungi leading nosocomial infections was investigated. Medilox® device uses salt, water and electricity and electrolyzes water. Medilox® device is calibrated according to the instructions of the producer to produce electrolyzed water at pH 6 including 80 ppm chlorine. End product is monitorized by pH test kit based on a color scale.

Six American Type Culture Collection (ATCC) strains (*Acinetobacter baumannii* 19606, *Escherichia coli* 25922, *Enterococcus faecalis* 29212, *Klebsiella pneumoniae* 254988, *Pseudomonas aeruginosa* 27853, *Staphylococcus aureus* 29213), eight multidrug-resistant bacteria isolated from different clinical samples (*Acinetobacter baumannii*, *Escherichia coli*, *vancomycin*-*resistant Enterococcus faecium*, *Klebsiella pneumoniae*, *Pseudomonas aeruginosa*, *methicillin*-*resistant Staphylococcus aureus*, *Bacillus subtilis*, *Myroides* spp.), yeasts (*Candida albicans*, *Candida tropicalis*, *Candida parapsilosis*, *Candida glabrata*, *Candida krusei*, *Candida lusitaniae*, *Trichosporon* spp.), and molds (*Aspergillus fumigatus*, *Aspergillus flavus*, *Aspergillus niger*) were used to evaluate in-vitro activity of different concentrations (1/1, 1/2, 1/5, 1/10, 1/20, 1/50, 1/100) and different contact times (1, 2, 5, 10, 30 min) of super-oxidized water by using qualitative suspension test method [2,10,11]. Dey-Engley Neutralizing Broth (Sigma-Aldrich, USA), (casein enzymatic hydrolyzate 5 g/l, yeast extract 2.5 g/l, dexros 10 g/l Sodium thiosulfate 1 g/l, sodium bisulfite 2.5 g/l, lecithin 7 g/l, Polysorbate 80 5 g/l, and bromacresol purple 0.02 g/l) was used as neutralizing agent. Bacteria were subcultured onto Tryptic Soy Agar (TSA, Oxoid, UK) and fungi subcultured onto Sabouraud Dextrose Agar (SDA). Cultures were incubated at 25 or 37°C for 24-72 hours according to the type of microorganism. Microorganism suspensions were prepared from 24 hours cultures of bacteria in Tryptic Soy Broth (TSB) by adjusting 0.5 McFarland turbidity standard (10^8^ CFU/ml). For yeasts and molds, suspensions were adjusted to 4 McFarland turbidity standard to achieve 12 × 10^6^ CFU/ml [12-14]. Ten micro liters from each microorganism suspension was added into the tubes containing 1000 μl disinfectant at different concentrations. The tubes were allowed to stand 1-2-5-10-30 minutes. When the exposure time is over, 100 μl of the mixture was added into another tube containing 900 μl neutralizing agent and 10 μl of the new mixture were spread onto TSA or SDA. Absence of growth on Petri dishes after 48-72 hours of incubation at relevant temperatures was interpreted as bactericidal or fungicidal activity. Microorganism suspensions without disinfectant were used as growth control and were spread onto agars following mixing with neutralizing agent.

## Results

Medilox® super-oxidized water was found to be effective against all standard strains and all clinical isolates tested at a dilution of 1/1 and exposure time of 1 minute. In addition, it has been found to be effective on ATCC and all other clinical isolates except VRE in a dilution of 1/2 within 1 minute and the other durations of exposure. It has been found to be most effective on *E. coli* isolates in a dilution of 1/5 (Tables 1 and 2).

**Table 1:** Effects of Medilox® super-oxidized on the growth of ATCC bacteria isolates

	**Medilox® [dilution rate]**						
		**1/1**	**1/2**	**1/5**	**1/10**	**1/50**	**1/100**
***Acinetobacter baumannii *** **19606**	1 min.	-	-	+	+	+	+
	2 min.	-	-	+	+	+	+
	5 min.	-	-	+	+	+	+
	10 min.	-	-	+	+	+	+
	30 min.	-	-	-	+	+	+
***E. coli *** **25922**	1 min.	-	-	+	+	+	+
	2 min.	-	-	+	+	+	+
	5 min.	-	-	-	+	+	+
	10 min.	-	-	-	+	+	+
	30 min.	-	-	-	+	+	+
***Enterococcus faecalis *** **29212**	1 min.	-	-	+	+	+	+
	2 min.	-	-	+	+	+	+
	5 min.	-	-	+	+	+	+
	10 min.	-	-	+	+	+	+
	30 min.	-	-	+	+	+	+
***Klebsiella pneumoniae *** **254988**	1 min.	-	-	+	+	+	+
	2 min.	-	-	+	+	+	+
	5 min.	-	-	+	+	+	+
	10 min.	-	-	+	+	+	+
	30 min.	-	-	+	+	+	+
***Pseudomonas aeruginosa *** **27853**	1 min.	-	-	+	+	+	+
	2 min.	-	-	+	+	+	+
	5 min.	-	-	+	+	+	+
	10 min.	-	-	+	+	+	+
	30 min.	-	-	+	+	+	+
***Staphylococcus aureus *** **29213**	1 min.	-	-	+	+	+	+
	2 min.	-	-	+	+	+	+
	5 min.	-	-	+	+	+	+
	10 min.	-	-	+	+	+	+
	30 min.	-	-	-	+	+	+

[-]: Growth was not observed, [+]: Growth was observed.

**Table 2:** Effects of Medilox® super-oxidized on the growth of clinical bacteria isolates

	**Medilox® [dilution rate]**						
		**1/1**	**1/2**	**1/5**	**1/10**	**1/50**	**1/100**
***Acinetobacter baumannii***	1 min.	-	-	+	+	+	+
	2 min.	-	-	+	+	+	+
	5 min.	-	-	+	+	+	+
	10 min.	-	-	+	+	+	+
	30 min.	-	-	-	+	+	+
***E. coli***	1 min.	-	-	+	+	+	+
	2 min.	-	-	+	+	+	+
	5 min.	-	-	-	+	+	+
	10 min.	-	-	-	+	+	+
	30 min.	-	-	-	+	+	+
**VRE**	1 min.	-	+	+	+	+	+
	2 min.	-	+	+	+	+	+
	5 min.	-	+	+	+	+	+
	10 min.	-	+	+	+	+	+
	30 min.	-	+	+	+	+	+
***Klebsiella pneumoniae***	1 min.	-	-	+	+	+	+
	2 min.	-	-	+	+	+	+
	5 min.	-	-	+	+	+	+
	10 min.	-	-	+	+	+	+
	30 min.	-	-	+	+	+	+
***Pseudomonas aeruginosa***	1 min.	-	-	+	+	+	+
	2 min.	-	-	+	+	+	+
	5 min.	-	-	+	+	+	+
	10 min.	-	-	+	+	+	+
	30 min.	-	-	+	+	+	+
***Staphylococcus aureus***	1 min.	-	-	+	+	+	+
	2 min.	-	-	+	+	+	+
	5 min.	-	-	+	+	+	+
	10 min.	-	-	+	+	+	+
	30 min.	-	-	-	+	+	+
***Bacillus subtilus***	1 min.	-	-	+	+	+	+
	2 min.	-	-	+	+	+	+
	5 min.	-	-	+	+	+	+
	10 min.	-	-	+	+	+	+
	30 min.	-	-	-	+	+	+
***Myroide*** **s spp.**	1 min.	-	-	+	+	+	+
	2 min.	-	-	+	+	+	+
	5 min.	-	-	+	+	+	+
	10 min.	-	-	+	+	+	+
	30 min.	-	-	-	+	+	+

[-]: Growth was not observed, [+]: Growth was observed.

Medilox® super-oxidized water was found to be effective against yeasts at a dilution of 1/1 and exposure time of 1 minute. It has been found to be effective in a dilution of 1/2 for *C. kruse and C. lusitaniae* with 5 minutes of exposure time, by using the same proportion of dilution, 2 minutes of exposure time has been needed for *Trichosporon* spp. and 1 minute of exposure time for the others (Table 3). Whereas required exposure time was ≥ 2 minutes for *Aspergillus flavus* and ≥ 5 minutes for *A. fumigatus* and *A. niger* at 1/1 dilution (Table 4).

**Table 3:** Effects of Medilox® super-oxidized on the growth of yeast isolates

	**Medilox® [dilution rate]**						
		**1/1**	**1/2**	**1/5**	**1/10**	**1/50**	**1/100**
***Candida albicans***	1 min.	-	-	+	+	+	+
	2 min.	-	-	+	+	+	+
	5 min.	-	-	+	+	+	+
	10 min.	-	-	+	+	+	+
	30 min.	-	-	-	+	+	+
***Candida tropicalis***	1 min.	-	-	+	+	+	+
	2 min.	-	-	+	+	+	+
	5 min.	-	-	+	+	+	+
	10 min.	-	-	+	+	+	+
	30 min.	-	-	-	+	+	+
***Candida parapsilosis***	1 min.	-	-	+	+	+	+
	2 min.	-	-	+	+	+	+
	5 min.	-	-	-	+	+	+
	10 min.	-	-	-	+	+	+
	30 min.	-	-	-	+	+	+
***Candida glabrata***	1 min.	-	-	+	+	+	+
	2 min.	-	-	+	+	+	+
	5 min.	-	-	+	+	+	+
	10 min.	-	-	+	+	+	+
	30 min.	-	-	-	+	+	+
***Candida krusei***	1 min.	-	+	+	+	+	+
	2 min.	-	+	+	+	+	+
	5 min.	-	-	+	+	+	+
	10 min.	-	-	+	+	+	+
	30 min.	-	-	+	+	+	+
***Candida lusitaniae***	1 min.	-	+	+	+	+	+
	2 min.	-	+	+	+	+	+
	5 min.	-	-	+	+	+	+
	10 min.	-	-	+	+	+	+
	30 min.	-	-	+	+	+	+
***Trichosporon spp.***	1 min.	-	+	+	+	+	+
	2 min.	-	-	+	+	+	+
	5 min.	-	-	+	+	+	+
	10 min.	-	-	+	+	+	+
	30 min.	-	-	-	+	+	+

[-]: Growth was not observed, [+]: Growth was observed.

**Table 4:** Effects of Medilox® super-oxidized on the growth of mold isolates

	**Medilox® [dilution rate]**						
		**1/1**	**1/2**	**1/5**	**1/10**	**1/50**	**1/100**
***Aspergillus fumigatus***	1 min.	+	+	+	+	+	+
	2 min.	+	+	+	+	+	+
	5 min.	-	-	+	+	+	+
	10 min.	-	-	+	+	+	+
	30 min.	-	-	-	+	+	+
***Aspergillus flavus***	1 min.	+	+	+	+	+	+
	2 min.	-	+	+	+	+	+
	5 min.	-	-	+	+	+	+
	10 min.	-	-	+	+	+	+
	30 min.	-	-	-	+	+	+
***Aspergillus niger***	1 min.	+	+	+	+	+	+
	2 min.	+	+	+	+	+	+
	5 min.	-	+	+	+	+	+
	10 min.	-	-	+	+	+	+
	30 min.	-	-	-	+	+	+

[-]: Growth was not observed, [+]: Growth was observed.

## Discussion

Disinfection is utmost important factor in the prevention of nosocomial infections. It has been known that failure in disinfection increases the morbidity, mortality, and treatment costs, whereas unnecessary disinfection procedures increase hospital expenses and select resistant microorganisms [15]. In order to avoid such risks, the first step in the hospital setting should be the selection of right disinfectants that have proven activity against broad spectrum of microorganisms. Relevant application method, right concentration, and required exposure time should be used [3]. Disinfection efficacy of electrolyzed water, which has been widely used on environment and water in recent years, is remarkable. It has many advantages such as not having toxic products, safety to the patients, the staff, and the environment, not harming human tissue, and low cost [8].

Super-oxidized water has been used in various industrial areas in our country in recent years. There are many international researches being performed to determine the efficacy of super-oxidized water. However, this study is one of the very few studies that will lead further studies investigating the activity of super-oxidized water on microorganisms causing nosocomial infections. Suspension tests are the most commonly used, inexpensive, easy to apply, reproducible first step tests to determine the activity of disinfectants [10]. In this study, we used suspension tests to evaluate activity of super-oxidized water against different types of bacteria and fungi causing hospital-acquired infections.

A disinfectant which can be used safely in hospital settings should be effective against bacteria, fungi, viruses, tubercle bacilli, and spores [3]. In this study, bacteria and fungi that will represent this flora and some other standard strains were used. VRE can lead hospital epidemics by contaminating medical devices and patient’s environment. Fast active surface disinfectants are necessary to be able to remove these microorganisms from the environment before they spread. In the present study it has been proven that super-oxidized water inactivated VRE in one minute. *Acinetobacter spp*. have also become an important problem especially in intensive care units due to their ability to survive long time on inanimate surfaces and ineffective disinfection procedures in hospitals. Our results have proved that super-oxidized water inactivated *A. baumannii* in one minute even at a dilution of ½.

Although fast lethal effect of super-oxidized water on bacteria, viruses, fungi and parasites is promising for surfaces and water disinfection systems, due to the lack of studies about this disinfectant, there is still suspicion for its usage [9]. Nishimura et al. [16] have reported that hand disinfection using super-oxidized water is 7.5% more effective than povidone iodine. Although it has a very fast antiseptic activity on hands, it has a major disadvantage on alcoholic hand rubs due to its long drying time. At the University of California, Landa et al. [17] used pure cultures of *Staphylococcus aureus, Escherichia coli, P. aeruginosa, Salmonella typhi,* and *Candida albicans* to evaluate in vitro antimicrobial efficacy testing of super-oxidized water. It has been found to be active on all bacteria and *C. albicans* tested. Sakurai et al. [18] have compared glutaraldehyde with super-oxidized water for endoscope disinfection against *Pseudomonas aeruginosa* and *Mycobacterium avium*. Endoscopes were immersed in electrolyzed water for 10 seconds and in comparison in gluteraldehyde at 5 and 10 minutes contact time. They have concluded that super-oxidized water is valuable and effective disinfectant for endoscopes. Choi et al. [19] have reported in the light of their study evaluating its activity on 25 bacterial strains and two fungi that Medilox® super-oxidized water can be used for disinfection of skin, instruments and surfaces. Choi [20], has reported that super- oxidized water is active against *Bacillus*, and *Candida* species besides various environmental flora bacteria and yeasts. Venkitanarayan et al. [21] have investigated the effectiveness of super-oxidized water against *Escherichia coli* O157: H7, *Salmonella enteritidis* and *Listeria monocytogenes* and reported that electrolyzed water can be used as an effective disinfectant provided that standardized application methods are used.

Recently, the use of super-oxidized water has attracted great interest in Japan. Tanaka et al. [22] have compared super-oxidized water with 2% Dialox -c and 3.8% formalin and reported that super-oxidized waterwas more effective than the other disinfectants. Nakayama, et al. [23] have proven that irrigation and disinfection of burn wounds using super-oxidized water may be helpful to prevent sepsis associated with burn injury. Vorobjev et al. [24] have reported that super-oxidized water is effective on spores and vegetative forms of spore forming bacteria as well as other gram positive and negative bacteria causing nosocomial infections. Fenner et al. [25] from University of Zurich have evaluated anti-microbial activity of super-oxidized water according to Veterinary German Association (DVG) Standard by using *Enterococcus faecium, Mycobacterium avium* subspecies *avium, Proteus mirabilis, Pseudomonas aeruginosa, Staphylococcus aureus,* and *Candida. albicans*. They have found that super-oxidized water was effective in 30 minutes on all the bacteria, and fungi tested. In the present study Medilox® super-oxidized water, was found to be effective on *P. aeruginosa, S. aureus*, VRE*. E.coli, K. pneumoniae*, and all types of ATCC bacteria, at 1/1 dilution in ≥1 minute.

Sterilox is another electrolyzed water which has been suggested for disinfection of dental water lines and endoscopes [26,27]. It has not been studied as a surface disinfectant against hospital microorganisms. Moreever, Rossi-Fedele et al. [28] have reported that the stability of this product was effected by the storage conditions and exposure to the sun.

Although there are many international studies investigating the efficacy of electrolyzed water, there are only a few studies evaluating the activity of electrolyzed water on fungi causing nosocomial infections. This study will guide further studies about the activity of super-oxidized water on fungal isolates. The incidence of *Candida* species has increased recently. *Candida* species has became the fourth most common cause of nosocomial bloodstream infection with the rate of 8 to 10%. The most commonly isolated fungal pathogen is *C. albicans* (59.8%), and is followed by other *Candida* species (18.6%) and *Aspergillus* species (1.3%) [4]. Different fungi isolated from different hospital environment may show different sensitivity to the most commonly used disinfectants. Therefore, determination of hospital microorganisms, and selection of relevant disinfectant active on these microorganisms is useful to prevent, and control hospital infections [29]. In our study, Medilox® super-oxidized water was found to be effective on yeast species in ≥ 1 minute; on *Aspergillus fumigatus*, *Aspergillus niger* ≥ 5 minutes, and on *Aspergillus flavus* ≥ 2 minutes at 1/1 dilution. In the light of the results of this study, super-oxidized water is considered as a surface disinfectant to prevent nosocomial fungal infections. *C. krusei,* and *C. parapsilosis* are important nosocomial agents which are spread in hospitals by health care workers’ hands, and lead epidemics. Our results have proved that super-oxidized water inactivates *C. krusei*, which is resistant to antifungal drugs, and *C. parapsilosis* in one minute, and at a ½ dilution. Qualitative suspension test which is one of the first step tests has been used to evaluate efficacy of super-oxidized water in this study. The results of this study proving the efficiency on wide variety of microorganisms causing hospital infections will ease the second, and third step studies.

In conclusion, our findings support that super-oxidized water produced by Medilox® disinfectant generator using water, salt, and electricity provides highly efficient disinfection. In the light of our results which have proven the in-vitro activity of Medilox® super-oxidized water on bacterial, and fungal isolates with different resistance patterns, we believe that super-oxidized water can be used efficiently to prevent hospital-acquired infections provided that further efficacy studies are done, and validated application methods are used.

## Competing interests

We declare that they have no competing interests.

## Authors' contributions

MG is Project Manager, *(He is worked in Samsun Ondokuz Mayıs University, Now He is working in Istanbul University, Cerrahpasa Medical Faculty, in İstanbul)* project designer and general supervisor, Laboratory working, writing the manuscript. SE Project designer and general supervision of the research group. (*Project co-Manager*). AK Laboratory supervisor, Analysis and interpretation of data and control of article. NU carried out the all laboratory studies *(He is worked in Samsun Ondokuz Mayis University, Now He is working* Samsun Maternity and Children's Hospital Laboratory of Microbiology in Samsun*).* KY collection of bacteria strain and writing the manuscript. HO carried out the some laboratory studies. AS collection of fungi and yeast. All authors read and approved the final manuscript*.*
